# Reference ranges for cardiac structure and function using cardiovascular magnetic resonance (CMR) in Caucasians from the UK Biobank population cohort

**DOI:** 10.1186/s12968-017-0327-9

**Published:** 2017-02-03

**Authors:** Steffen E. Petersen, Nay Aung, Mihir M. Sanghvi, Filip Zemrak, Kenneth Fung, Jose Miguel Paiva, Jane M. Francis, Mohammed Y. Khanji, Elena Lukaschuk, Aaron M. Lee, Valentina Carapella, Young Jin Kim, Paul Leeson, Stefan K. Piechnik, Stefan Neubauer

**Affiliations:** 1 William Harvey Research Institute, NIHR Cardiovascular Biomedical Research Unit at Barts, Queen Mary University of London, Charterhouse Square, London, EC1M 6BQ UK; 2 Division of Cardiovascular Medicine, Radcliffe Department of Medicine, University of Oxford, Level 6, West Wing, John Radcliffe Hospital, Headington, Oxford, OX3 9DU UK; 3 Department of Radiology, Severance Hospital, Yonsei University College of Medicine, 50-1 Yonsei-ro, Seodaemun-gu, Seoul, 03722 South Korea

**Keywords:** Cardiovascular magnetic resonance, Reference values, Ventricular function, Atrial function

## Abstract

**Background:**

Cardiovascular magnetic resonance (CMR) is the gold standard method for the assessment of cardiac structure and function. Reference ranges permit differentiation between normal and pathological states. To date, this study is the largest to provide CMR specific reference ranges for left ventricular, right ventricular, left atrial and right atrial structure and function derived from truly healthy Caucasian adults aged 45–74.

**Methods:**

Five thousand sixty-five UK Biobank participants underwent CMR using steady-state free precession imaging at 1.5 Tesla. Manual analysis was performed for all four cardiac chambers. Participants with non-Caucasian ethnicity, known cardiovascular disease and other conditions known to affect cardiac chamber size and function were excluded. Remaining participants formed the healthy reference cohort; reference ranges were calculated and were stratified by gender and age (45–54, 55–64, 65–74).

**Results:**

After applying exclusion criteria, 804 (16.2%) participants were available for analysis. Left ventricular (LV) volumes were larger in males compared to females for absolute and indexed values. With advancing age, LV volumes were mostly smaller in both sexes. LV ejection fraction was significantly greater in females compared to males (mean ± standard deviation [SD] of 61 ± 5% vs 58 ± 5%) and remained static with age for both genders. In older age groups, LV mass was lower in men, but remained virtually unchanged in women. LV mass was significantly higher in males compared to females (mean ± SD of 53 ± 9 g/m^2^ vs 42 ± 7 g/m^2^). Right ventricular (RV) volumes were significantly larger in males compared to females for absolute and indexed values and were smaller with advancing age. RV ejection fraction was higher with increasing age in females only. Left atrial (LA) maximal volume and stroke volume were significantly larger in males compared to females for absolute values but not for indexed values. LA ejection fraction was similar for both sexes. Right atrial (RA) maximal volume was significantly larger in males for both absolute and indexed values, while RA ejection fraction was significantly higher in females.

**Conclusions:**

We describe age- and sex-specific reference ranges for the left ventricle, right ventricle and atria in the largest validated normal Caucasian population.

**Electronic supplementary material:**

The online version of this article (doi:10.1186/s12968-017-0327-9) contains supplementary material, which is available to authorized users.

## Background

Quantitative assessment of the cardiac chambers is vital for the determination of pathological states in cardiovascular disease. Intrinsic to this is knowledge of reference values for morphological and functional cardiovascular parameters specific to cardiovascular magnetic resonance (CMR), the most advanced tool for imaging the human heart. CMR has rapidly evolved towards faster and more detailed imaging methods limiting the generalisability of earlier results from relatively small studies [1–4]. More recent studies detailing “normal” ranges for CMR are limited by inclusion of individuals with cardiovascular risk factors such as obesity, diabetes and current smokers in their reference cohort [5, 6].

The UK Biobank is amongst the world’s largest population-based prospective studies, established to investigate the determinants of disease in middle and old age [7]. In addition to the collection of extensive baseline questionnaire data, biological samples and physical measurements, CMR is utilized to provide cardiovascular imaging-derived phenotypes [8].

Based on the UK Biobank participant demographics and health status in ~5000 consecutive participants from the early phase of CMR [8, 9], we aim to select validated normal healthy Caucasian participants in order to establish reference values for left ventricular, right ventricular, left atrial and right atrial structure and function.

## Methods

### Study population

CMR examinations of 5,065 consecutive UK Biobank participants were assessed. Participants with non-Caucasian ethnicity, known cardiovascular disease, hypertension, respiratory disease, diabetes mellitus, hyperlipidaemia, haematological disease, renal disease, rheumatological disease, malignancy, symptoms of chest pain or dyspnoea, current- or ex-tobacco smokers, those taking medication for diabetes, hyperlipidaemia or hypertension and those with BMI ≥30 kg/m^2^ [10] were excluded from the analysis. In order to create evenly distributed age-decade groups (45–54, 55–64, 65–74), all participants older than 74 years were also excluded from the cohort. (See Appendix 1 for the full list of exclusions).

### CMR protocol

The full CMR protocol in the UK Biobank has been described in detail elsewhere [9]. In brief, all CMR examinations were performed in Cheadle, United Kingdom, on a clinical wide bore 1.5 Tesla scanner (MAGNETOM Aera, Syngo Platform VD13A, Siemens Healthcare, Erlangen, Germany).

Assessment of cardiac function was performed based on combination of several cine series: long axis cines (horizontal long axis – HLA, vertical long axis – VLA, and left ventricular outflow tract –LVOT cines, both sagittal and coronal) and a complete short axis stack covering the left ventricle (LV) and right ventricle (RV) were acquired at one slice per breath hold. All acquisitions used balanced steady-state free precession (bSSFP) with typical parameters (subject to standard radiographer changes to planning), as follows: TR/TE = 2.6.1.1 ms, flip angle 80°, Grappa factor 2, voxel size 1.8 mm × 1.8 mm × 8 mm (6 mm for long axis). The actual temporal resolution of 32 ms was interpolated to 50 phases per cardiac cycle (~20 ms). No signal or image filtering was applied besides distortion correction.

### Image analysis

Manual analysis of LV, RV, LA and RA were performed across two core laboratories based in London and Oxford, respectively. Standard operating procedures for analysis of each chamber were developed and approved prior to study commencement. CMR scans were analysed using cvi^42^ post-processing software (Version 5.1.1, Circle Cardiovascular Imaging Inc., Calgary, Canada).

In each CMR examination, the end-diastolic phase was selected as the first phase of the acquisition. Observers selected the end-systolic phase by determining the phase in which the LV intra-cavity blood pool was at its smallest by visual assessment at the mid-ventricular level. LV endocardial and epicardial borders were manually traced in both the end-diastolic and end-systolic phases in the short-axis view. In both end-diastole and end-systole, the most basal slice for the LV was selected when at least 50% of the LV blood pool was surrounded by myocardium. In order to reduce observer variability, LV papillary muscles were included as part of LV end-diastolic volume and end-systolic volume, and excluded from LV mass. As an internal quality control measure, the LV mass values in both diastole and systole were checked to ensure they are almost identical. In cases with significant discrepancy, the contours were reviewed and corrected through consensus group approach.

For the RV, endocardial borders were manually traced in end-diastole and end-systole in the short axis view. Volumes below the pulmonary valve were included. At the inflow tract, thin-walled structures without trabeculations were not included as part of the RV. RV end-diastolic and end-systolic phases were denoted to be the same as those for the LV. LV and RV stroke volumes were checked to ensure they were similar.

**Fig. 1:**
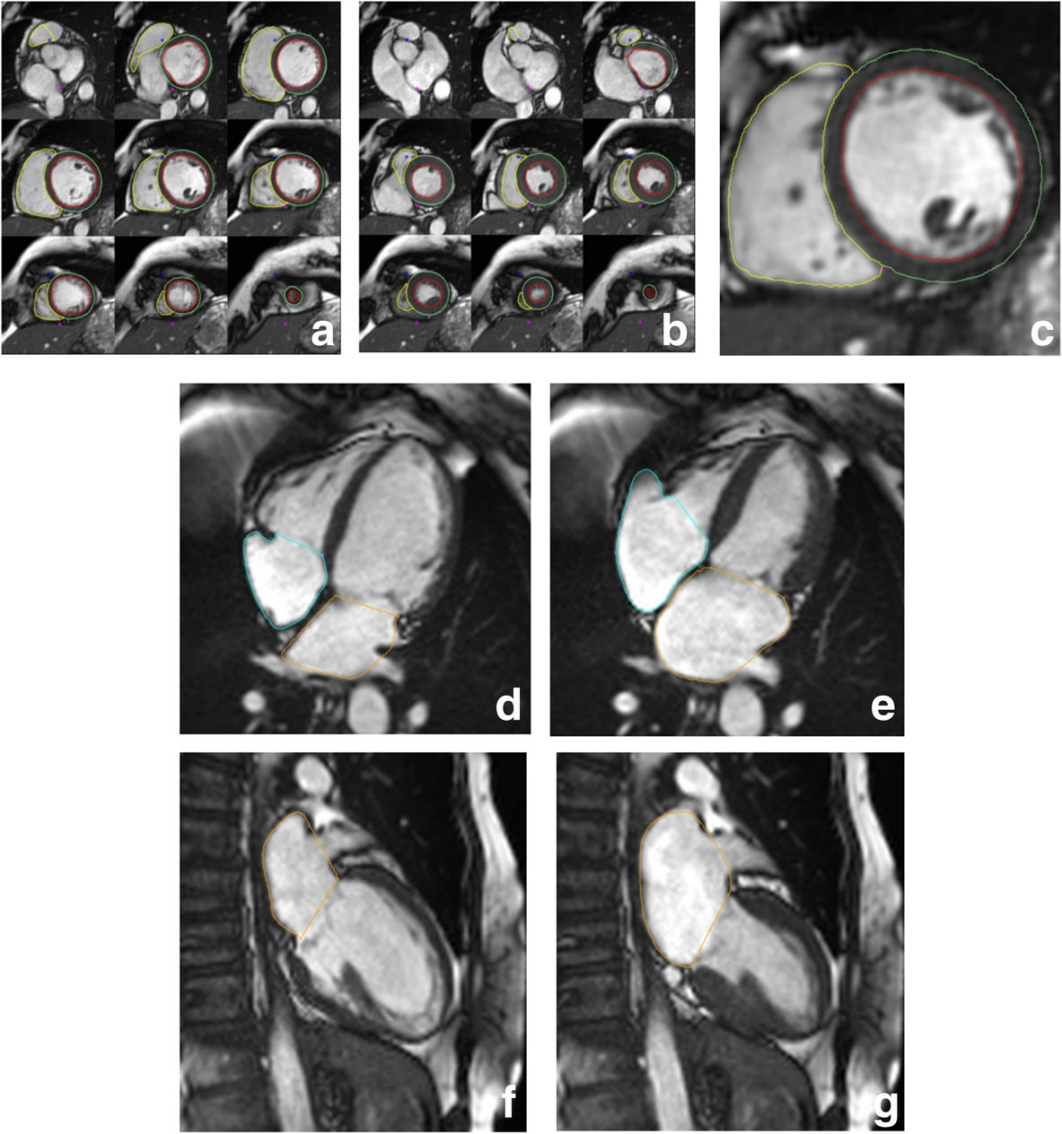
Examples of ventricular and atrial contours. The above panels are representative of analysis undertaken on each CMR examination. **a** and **b** demonstrate contouring of the left and right ventricle from base to apex at end-diastole and end-systole, respectively. **d** and **e** demonstrate contouring of the left and right atrium in the four-chamber view. **f** and **g** demonstrate contouring of the left atrium in the two-chamber view

LA and RA end-diastolic volume, end-systolic volume, stroke volume and ejection fraction were derived by manually tracing endocardial LA contours at end-systole (maximal LA area) and end-diastole (minimal LA area) in the HLA (4-chamber) view. For LA, the same measurements were also derived from the VLA (2-chamber) view and LA volumes were calculated according to the biplane area-length method. Example contours for all four cardiac chambers are provided in Fig. 1.

### Inter-observer and inter-centre quality assurance aspects

Image analysis was undertaken by a team of eight observers under guidance of three principal investigators. For all cases, analysts filled in progress sheets to monitor any problems in evaluation of CMR data, with any problematic cases flagged, such as a significant discrepancy (defined as more than 10% difference). For such flagged cases all contours and images were reviewed looking for presence of artefacts or slice location problems, operator error or evidence of pathology, such as significant shunt or valve regurgitation. These cases were discussed in regular inter-centre meetings by teleconferencing with respective decisions closed by consensus of at least three team members with relevant knowledge. The team included two biomedical engineers, one radiologist, two career image analysts and six cardiologists. The quality assessment outputs were subject to formal ontological analysis [11]. Inter- and intra-observer variability between analysts for atrial and ventricular measurements was assessed by analysis of fifty, randomly-selected CMR examinations, repeated after a one-month interval.

### Statistical analysis

All data is presented as mean ± standard deviation unless stated otherwise. Continuous variables were visually assessed for normality using histograms and Q-Q plots. Independent sample Student’s *t-test* was used to compare the mean values of CMR parameters between men and women. Outliers were defined *a priori* as CMR measurements more than three interquartile ranges below the first quartile or above the third quartile and removed from analysis. Mean values for all cardiac parameters are presented by gender and decade (45–54, 55–64, 65–74). Reference ranges for measured (volume, mass) and derived (ejection fraction) data are defined as the 95% prediction interval which is calculated by mean ± t_0.975, n-1_ (√(n + 1)/n) (standard deviation) [12]. Absolute values were indexed to body surface area (BSA) using the DuBois and DuBois formula [13].

The normal ranges for the whole cohort (aged 45–74) were defined as the range where the measured value fell within the 95% prediction interval for the whole cohort regardless of age decade. The borderline zone was defined as the upper and lower ranges where the measured value lay outside the 95% prediction interval for at least one age group. The abnormal zone was defined as the upper and lower ranges where the measured values were outside the 95% prediction interval for any age group.

Pearson’s correlation coefficient was used to assess the impact of age on ventricular and atrial volumes and function. Intra-class correlation coefficients (ICC) were calculated to assess inter- and intra-observer variability, and were visually assessed using Bland-Altman plots [14]. Two-way ICC (2,1) was computed for inter-observer ICCs, to reflect the fact that a sample of cases and a sample of raters were observed, whilst a one-way ICC (1,1) was computed for intra-observer ICC [15]. A *p*-value <0.05 was considered statistically significant for all tests performed. Statistical analysis was performed using R (version 3.3.0) Statistical Software [16].

## Results

**Fig. 2:**
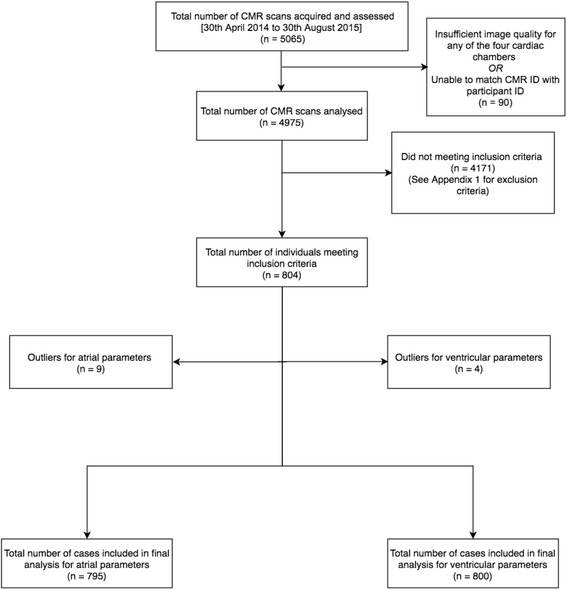
Case selection flowchart

A total of 5,065 CMR examinations underwent manual image analysis. 90 subjects were excluded as either the CMR data was of insufficient quality or the CMR identifier did not match the participant identifier. Of the remaining 4,975, 804 (16.2%) met the inclusion criteria. The breakdown of the number of participants meeting individual exclusion criterion is available in Appendix 1. The mean age of the cohort was 59 ± 7 (range 45–74) years. Upon removing outliers, a total of 800 participants (368 males, 432 females) were included in the ventricular analysis and 795 participants (363 male, 432 female) in the atrial analysis (Fig. 2). Baseline characteristics for all participants are provided in Table 1. A summary of CMR parameters stratified by gender is presented in Appendix 2, Tables 13 and 14. The association between CMR parameters and age stratified by gender is included in Appendix 2, Tables 14 and 15.

**Table 1:** Baseline Characteristics

	Age groups (years)		
	45-54	55-64	65-74
Number of participants	240	333	231
Age (years)	51 (±2)	59 (±3)	68 (±2)
Male gender (n(%))	110 (45.8%)	159 (47.7%)	102 (44.2%)
Systolic blood pressure (mmHg)	126 (±14)	133 (±17)	137 (±17)
Diastolic blood pressure (mmHg)	76 (±8)	78 (±9)	77 (±9)
Heart rate (bpm)	67 (±10)	69 (±12)	70 (±11)
Weight (kg)	71 (±13)	71 (±12)	69 (±11)
Height (cm)	171 (±9)	170 (±9)	168 (±9)
Body surface area (m^2^)	1.82 (±0.20)	1.82 (±0.19)	1.78 (±0.18)
Body mass index (kg/m^2^)	24.2 (±2.9)	24.4 (±2.7)	24.4 (±2.8)

All continuous values are reported in mean ± standard deviation (SD), while categories are reported as number (percentage)

*LV* left ventricle, *RV* right ventricle, *EDV* end-diastolic volume, *ESV* end-systolic volume, *SV* stroke volume, *EF* ejection fraction; indexed, absolute values divided by body surface area

CMR left ventricular, right ventricular, left atrial and right atrial reference ranges are provided in a traffic light format for males and females for the whole cohort regardless of their age groups for both absolute and indexed values in numerical format (Tables 2, 3, 4 and 5). These tables are also presented together in a user-friendly poster format for clinical use which is available in Additional file 1. Age-specific reference ranges are also provided in ‘look-up’ tables for those measured CMR values in the borderline (yellow) zone. (Tables 6, 7, 8, 9)

**Table 2:**
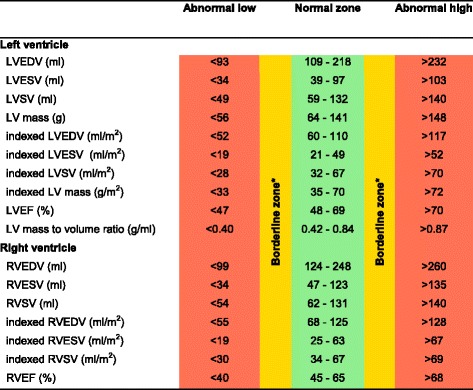
Ventricular reference range for Caucausian men

Abnormal low and high refer to the lower and upper reference limits, respectively. They are defined as measurements which lie outside the 95% prediction interval at all age groups

^a^Borderline zone values should be looked up in the age-specific tables. The borderline zone was defined as the upper and lower ranges where the measured value lay outside the 95% prediction interval for at least one age group

*LV* left ventricle, *RV* right ventricle, *EDV* end-diastolic volume, *ESV* end-systolic volume, *SV* stroke volume, *EF* ejection fraction; indexed, absolute values divided by body surface area

**Table 3:**
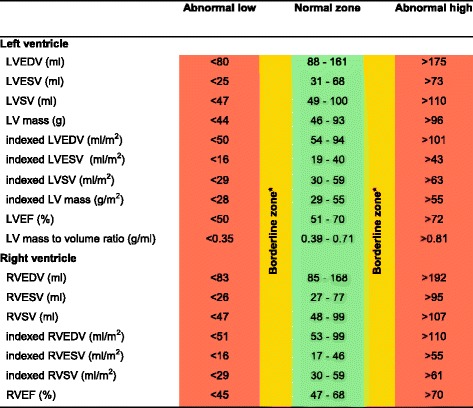
Ventricular reference range for Caucausian women

Abnormal low and high refer to the lower and upper reference limits, respectively. They are defined as measurements which lie outside the 95% prediction interval at all age groups

^a^Borderline zone values should be looked up in the age-specific tables. The borderline zone was defined as the upper and lower ranges where the measured value lay outside the 95% prediction interval for at least one age group

*LV* left ventricle, *RV* right ventricle, *EDV* end-diastolic volume, *ESV* end-systolic volume, *SV* stroke volume, *EF* ejection fraction; indexed, absolute values divided by body surface area

**Table 4:**
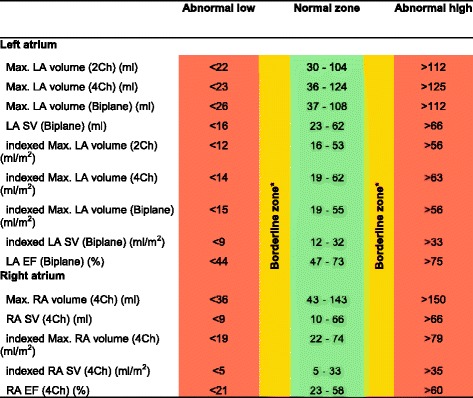
Atrial reference range for Caucausian men

Abnormal low and high refer to the lower and upper reference limits, respectively. They are defined as measurements which lie outside the 95% prediction interval at all age groups

^a^Borderline zone values should be looked up in the age-specific tables. The borderline zone was defined as the upper and lower ranges where the measured value lay outside the 95% prediction interval for at least one age group

*LA* left atrium, *RA* right atrium, *SV* stroke volume, *EF* ejection fraction, *2Ch* two-chamber, *4Ch* four-chamber, *Biplane* derived from four-chamber and two-chamber views; indexed, absolute values divided by body surface area

**Table 5:**
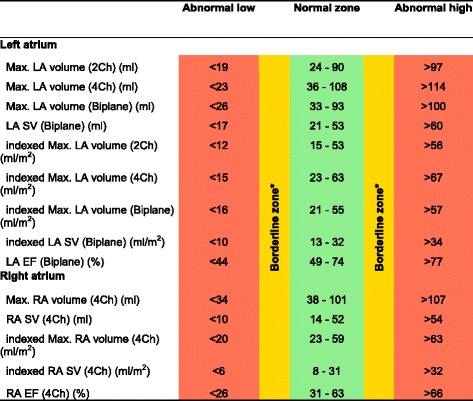
Atrial reference range for Caucausian women

Abnormal low and high refer to the lower and upper reference limits, respectively. They are defined as measurements which lie outside the 95% prediction interval at all age groups

^a^Borderline zone values should be looked up in the age-specific tables. The borderline zone was defined as the upper and lower ranges where the measured value lay outside the 95% prediction interval for at least one age group

*LA* left atrium, *RA* right atrium, *SV* stroke volume, *EF* ejection fraction, *2Ch* two-chamber, *4Ch* four-chamber, *Biplane* derived from four-chamber and two-chamber views; indexed, absolute values divided by body surface area

**Table 6:**
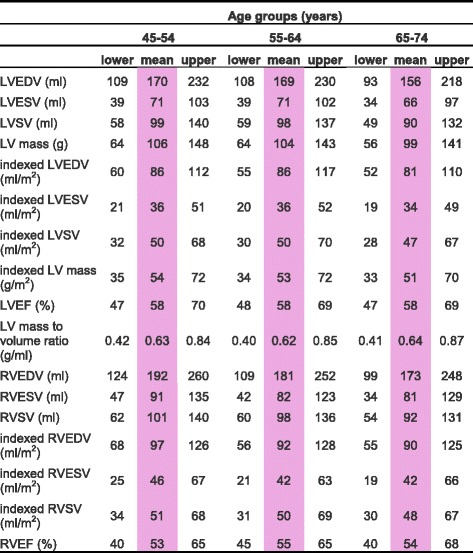
Age-specific ventricular reference ranges for Caucausian men

Male left and right atrial reference ranges detailing mean, lower reference limit and upper reference limit by age group. Reference limits are derived by the upper and lower bounds of the 95% prediction interval for each parameter at each age group

*LV* left ventricle, *RV* right ventricle, *EDV* end-diastolic volume, *ESV* end-systolic volume, *SV* stroke volume, *EF* ejection fraction; indexed, absolute values divided by body surface area

**Table 7:**
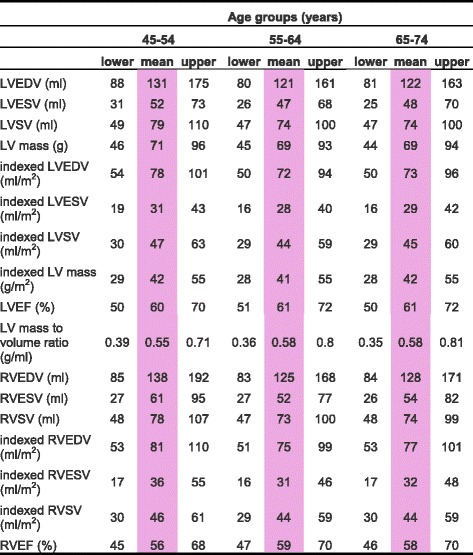
Age-specific ventricular reference ranges for Caucausian women

Male left and right atrial reference ranges detailing mean, lower reference limit and upper reference limit by age group. Reference limits are derived by the upper and lower bounds of the 95% prediction interval for each parameter at each age group

*LV* left ventricle, *RV* right ventricle, *EDV* end-diastolic volume, *ESV* end-systolic volume, *SV* stroke volume, *EF* ejection fraction; indexed, absolute values divided by body surface area

**Table 8:**
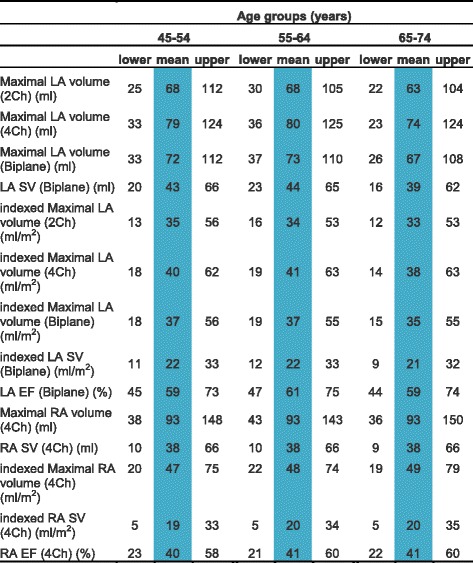
Age-specific atrial reference ranges for Caucausian men

Male left and right atrial reference ranges detailing mean, lower reference limit and upper reference limit by age group. Reference limits are derived by the upper and lower bounds of the 95% prediction interval for each parameter at each age group

*LA* left atrium, *RA* right atrium, *SV* stroke volume, *EF* ejection fraction, *2Ch* two-chamber, *4Ch* four-chamber, *Biplane* derived from four-chamber and two-chamber views; indexed, absolute values divided by body surface area

**Table 9:**
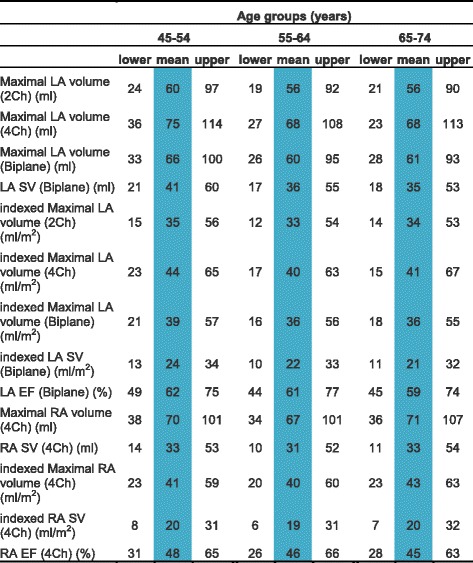
Age-specific atrial reference ranges for Caucausian women

Male left and right atrial reference ranges detailing mean, lower reference limit and upper reference limit by age group. Reference limits are derived by the upper and lower bounds of the 95% prediction interval for each parameter at each age group

*LA* left atrium, *RA* right atrium, *SV* stroke volume, *EF* ejection fraction, *2Ch* two-chamber, *4Ch* four-chamber, *Biplane* derived from four-chamber and two-chamber views; indexed, absolute values divided by body surface area

### Left ventricle

LV end-diastolic volume and LV end-systolic volume were significantly larger in males (LV EDV: absolute = 166 ± 32 ml, indexed = 85 ± 15 ml; LV ESV: absolute = 69 ± 16 ml, indexed = 36 ± 8 ml) compared to females (LV EDV: absolute = 124 ± 21 ml, indexed = 74 ± 12 ml; LV ESV: absolute = 49 ± 11 ml, indexed = 29 ± 6 ml) for both absolute and indexed values. (Appendix 2, Table 12) In men, LV end-diastolic volumes and stroke volumes were lower with older age for both absolute and indexed values. (Appendix 2, Table 14) In women, LV end-diastolic volume, end-systolic volume and stroke volume were smaller with advancing age for absolute and indexed values. LV ejection fraction was significantly greater in females (61 ± 5%) compared to males (58 ± 5%). LV ejection fraction demonstrated no correlation with age in neither males nor females. LV mass was significantly higher in males (103 ± 21 g) compared to females (70 ± 13 g). Upon normalization for body surface area, LV mass did not change significantly with age in either gender. In females, LV mass to end-diastolic volume ratio, a measure of distinct patterns of anatomical adaptations [17], increased significantly (*r* = 0.14, *p* <0.01) with age; this was not demonstrated in males.

### Right ventricle

RV end-diastolic volume and RV end-systolic volume were significantly larger in males (RV EDV: absolute = 182 ± 36 ml, indexed = 93 ± 17 ml; RV ESV: absolute = 85 ± 22 ml, indexed = 43 ± 11 ml) compared to females (RV EDV: absolute = 130 ± 24 ml, indexed = 77 ± 13 ml; RV ESV: absolute = 55 ± 15 ml, indexed = 33 ± 9 ml) for both absolute and indexed values. Both RV end-diastolic volume and end-systolic volume were lower in older age groups in males and females for absolute and indexed values. RV ejection fraction was significantly higher in females (58 ± 6%) compared to males (54 ± 6%). RV ejection fraction demonstrated a weak but significant positive correlation with advancing age in females only (r = 0.1, *p* < 0.05).

### Left and right atria

Left and right atrial reference ranges are presented in Tables 4, 5, 8 and 9. LA maximal volume and stroke volume, as determined by the biplane method, were significantly larger in males compared to females for absolute values (71 ± 19 vs 62 ± 17 ml) but not for BSA-indexed values (36 ± 9 vs 37 ± 10 ml). LA ejection fraction was almost identical (60% vs 61%) in males and females. Upon normalization for BSA, there was no change in left atrial volumes or function with age in men. In women, indexed LA stroke volume was significantly lower (r = −0.2, *p* < 0.001) with advancing age.

RA maximal volume and stroke volume were significantly larger in males (RA absolute maximal volume = 93 ± 27 ml, RA absolute stroke volume = 38 ± 14 ml) compared to females (RA absolute maximal volume = 69 ± 17 ml, RA absolute stroke volume = 32 ± 10 ml) for absolute values; upon indexing for BSA, this effect was seen for RA maximal volume only (48 ± 14 vs 41 ± 10 ml). RA ejection fraction was significantly higher (46% vs 41%, *p* < 0.001) in females compared to males. Upon normalization for BSA, there was no change in right atrial volumes or function with age in males or females.

### Intra- and inter-observer variability

**Fig. 3:**
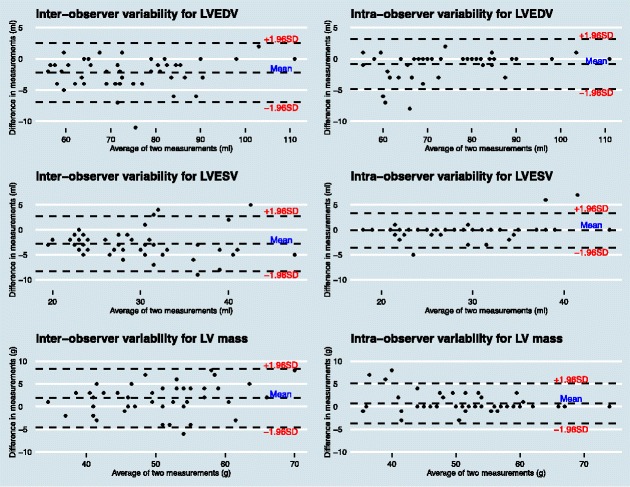
Exemplar Bland-Altman plots for inter- and intra-observer variability of left ventricular parameters

**Fig. 4:**
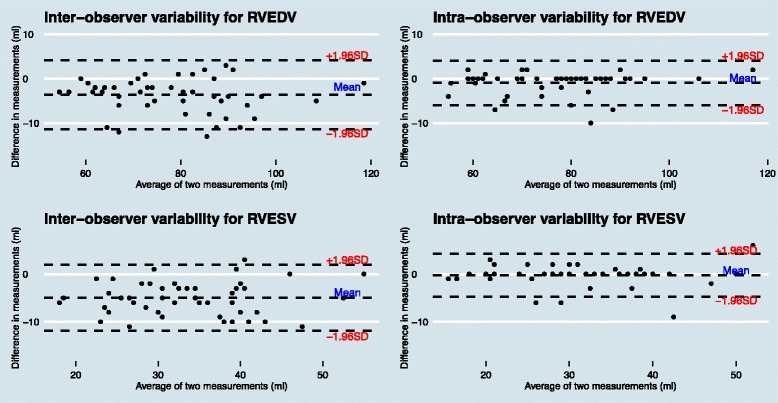
Exemplar Bland-Altman plots for inter- and intra-observer variability of right ventricular parameters

**Fig. 5:**
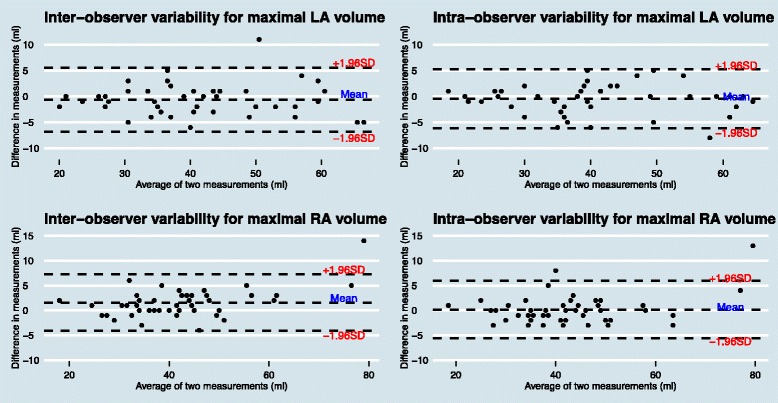
Exemplar Bland-Altman plots for inter- and intra-observer variability of atrial parameters

Intra and inter-observer variability data is presented in Table 10 and as Bland-Altman plots (representative examples of all observers) in Appendix 3, Figures 3, 4 and 5. Good to excellent intra- and inter-observer variability was achieved for LV and RV end-diastolic volume, end-systolic volume and stroke volume and LA and RA maximal volume and stroke volume.

**Table 10:** Inter- and intra-observer variability

	Inter-observer ICC*	Intra-observer ICC range^a^
Ventricle		
LVEDV	0.97	0.98-1.00
LVESV	0.88	0.95-0.97
LVSV	0.92	0.91-0.98
LVEF	0.71	0.80-0.92
LV mass	0.92	0.97-0.97
LV mass to volume ratio	0.92	0.79-0.97
RVEDV	0.92	0.98-0.99
RVESV	0.77	0.90-0.97
RVSV	0.89	0.93-0.98
RVEF	0.64	0.78-0.95
Atrium		
Maximal LA volume	0.96	0.97-0.98
LASV	0.90	0.90-0.96
LAEF	0.64	0.75-0.93
Maximal RA volume	0.96	0.97-0.99
RASV	0.86	0.92-0.94
RAEF	0.75	0.84-0.88

*ICC* Intra-class correlation coefficient, *LV* left ventricle, *RV* right ventricle, *EDV* end-diastolic volume, *ESV* end-systolic volume, *SV* stroke volume, *EF* ejection fraction, *LA* left atrium, *RA* right atrium

*p-value < 0.001

^a^Range of all observers, *p*-value < 0.001

## Discussion

The present study provides clinically relevant age- and gender-specific CMR reference ranges in a traffic light system for the left ventricular, right ventricular, left atrial and right atrial chambers derived from a cohort of 804 Caucasian adults aged 45–74 strictly free from pathophysiological or environmental risk factors affecting cardiac structure or function at 1.5 Tesla.

Whilst determination of reference ranges for CMR has been performed by several previous studies, this work is novel for a number or reasons. Firstly, the substantially larger cohort with strict evidence to ensure participants are free of biological or environmental factors known to impact upon cardiac structure or function differentiates this study from its predecessors. Secondly, reference ranges for CMR parameters are detailed not only by gender but also by age decade, thereby providing increased granularity and clinical utility. Thirdly, previously described findings are reinforced, particularly with respect to age- and gender-related differences in ventricular and atrial parameters. Fourthly, in-depth data surrounding intra- and inter-observer variability is provided.

The validity of a reference range is dependent on a number of factors, including the number of observations available in order to determine the reference interval [12]. This study utilises 800 participants for derivation of left and right ventricular reference ranges. This is a substantial increase compared to the majority of previous studies describing ventricular reference ranges using the SSFP technique: Alfakih et al. [3] (*n* = 60), Hudsmith et al. [2] (*n* = 108), Maceira et al. [1] (*n* = 120) and similar to those published by the Framingham Heart Study group. Similarly, 795 participants are included for derivation of left and right atrial reference ranges. Although previous studies outlining atrial reference ranges have used differing techniques, again, all utilise substantially fewer participants: Sievers et al. [18] (*n* = 111), Hudsmith et al. [2] (*n* = 108), Maceira et al. [19, 20] (*n* = 120). Even a recent systematic review and meta-analysis of normal values for CMR in adults and children is based on smaller numbers than the normal reference ranges presented here [4]. A recently published paper by Gandy and colleagues presents LV reference ranges for 1,515 UK individuals scanned at 3 Tesla [21]. However, their study population includes participants with high plasma B type natriuretic peptide (BNP) levels and blood pressure >149/95 mmHg by design, thus, could not be considered strictly healthy. Le Van et al. describes ventricular and atrial reference values derived from 434 Caucasian adults with similar exclusion criteria to the present study [22]. However, their study examines a much younger cohort, aged 18 to 35 years, and thus the present study complements their findings by investigating an older age range.

Furthermore, this study complied with approved statistical recommendations on derivation of reference limits [12]. Data has been partitioned – dividing reference values by age and sex – in order to reduce variation. The distribution of the reference values was inspected and assessed for normality and values identified as outliers discarded as per our *a priori* definition.

A total of 5,065 CMR examinations of UK Biobank participants were analysed for this study. Utilising this large population sample permitted *a posteriori* (retrospective) selection of the reference sample, the preferred method when compiling reference values from healthy individuals [23]. Indeed, only 16% of the original sample were included in this study, with rule-out criteria extending beyond known cardiovascular disease to include traditional cardiovascular risk factors (diabetes mellitus, hypercholesterolaemia, hypertension, current- and ex-tobacco smokers, obesity), cardiovascular symptoms, current or previous cancer, stroke, respiratory, renal or haematological disease and use of certain pharmacological agents. In doing so, a robust definition of what constitutes “health” was created, permitting confidence that reference ranges for cardiovascular structure and function in CMR have been derived from an appropriately selected cohort. This contrasts to the LV reference values published from the Framingham Heart Study Offspring Cohort where the healthy reference group consisted of 47.5% of the total cohort, and exclusion criteria were a history of hypertension, history of use of antihypertensive medication, previous myocardial infarction and heart failure only. Similarly, in the RV reference values study published by the same group, the “healthy reference” cohort included participants with hypertension, diabetes, hypercholesterolaemia and those who were current tobacco smokers [6].

For the left ventricle, our findings that men demonstrated greater volumes and mass compared to females is consistent with both the CMR literature [4] and that derived from other imaging modalities [24, 25]. Our demonstration of decreasing LV end-diastolic and end-systolic volumes with advancing age is also consistent with previous findings. Values for LV end-diastolic volumes are similar to those described by Hudsmith [2], Kawel-Boehm [4] and the Framingham Offspring Cohort group. LV end-systolic volumes were larger, reflecting this study’s methodology of including papillary muscles as part of the LV cavity – the technique most commonly employed when analysing clinical CMR examinations. Consequently, LV ejection fraction mean values and reference intervals were lower than previously reported. Despite this, the finding of a marginally, but significantly, lower LV ejection fraction in men compared to women is consistent with other large cohorts, including the Framingham Offspring Cohort, the Dallas Heart Study cohort [26] and the Multi-Ethnic Study of Atherosclerosis (MESA) cohort [27], although the latter two studies utilised the older gradient-recalled echo sequences. Our study demonstrated no change in LV ejection fraction across age groups, this is consistent with studies across imaging modalities [28, 29]. LV mass, upon normalization for BSA, did not change significantly across age groups in either gender. This is consistent with findings from the MESA cohort, but differs from the Framingham Offspring cohort which demonstrated a significant decrease in BSA-normalised LV mass with age. Autopsy-derived data concerning LV mass in individuals free from hypertension and coronary artery disease and corrected for BSA corroborate findings from our study, suggesting no change in cardiac mass with ageing [30].

For the right ventricle, our findings that males exhibited greater absolute and indexed volumes than females and that volumes were lower with advancing age in both genders are consistent with previously published literature. We demonstrated a larger RV ejection fraction in women compared to men, this is corroborated by Alfakih [3] using both SSFP and gradient-recalled echo sequences and by Foppa and Arora in the Framingham Offspring cohort [6].

For the atrial chambers, no consensus exists regarding the measurement of atrial volumes [4]. In this study, the LA was contoured in the 4-chamber and 2-chamber views and volumes calculated according to the biplane area-length method. Only Hudsmith presented LA reference ranges utilising a similar method with values for LA ejection fraction being almost identical to those described in this study. For the RA, the most recent work regarding reference ranges has been produced by Maceira et al. [20] using three-dimensional modelling which has not been undertaken in this study. Despite different methodology, general findings regarding absolute values being greater in males compared to females and no significant effect of age on RA volumes were replicated in our larger study.

### Clinical utility

CMR measurements only provide meaningful information when compared to relevant reference values. However, comparison may be misleading if the CMR examination being considered does not adequately match the reference sample, particularly with regards to age and gender. It is known that cardiovascular disease predominantly affects individuals in middle- and old-age, and it is individuals in these age groups who most commonly undergo CMR examinations. Furthermore, atrial and ventricular structure and function do not remain static over time and undergo changes with age, even in those without evidence of cardiovascular disease. It is in this context that this study presents absolute and BSA-indexed CMR reference values for men and women at three different age groups: 45–54, 55–64 and 65–74.

### Intra- and inter-observer variability

For LV and RV end-diastolic volume, end-systolic volume and stroke volume and LA and RA maximal volume and stroke volume, excellent inter- and intra-observer variability was achieved. It is notable, but perhaps not unsurprising, that ICC for derived parameters (i.e. ejection fraction) fell in comparison to those values for directly measured parameters. This is consistent with previous studies examining variability in CMR analysis, such as Margossian et al. [31] and Teo et al. [32], which reported very high inter-observer ICC’s for measured parameters which fell markedly when assessing the ejection fraction.

### Study limitations

The reference intervals described were derived from a population of 45–74 year olds of Caucasian ethnicity and therefore may not be generalisable to other ethnic and age groups. As the UK Biobank Imaging project accumulates CMR imaging in up to 100,000 individuals in coming years, analysis of ethnicity effects will become feasible in due course. We included overweight participants with a BMI between 25 and 30 kg/m^2^ in our reference range analysis, even though previous CMR publications, including our own, have shown that obesity affects cardiac structure and function even in an otherwise healthy population [33, 34]. Our rationale for this inclusion was two-fold: firstly, we aligned our inclusion criteria related to BMI with the “Recommendations for Cardiac Chamber Quantification by Echocardiography in Adults: An Update from the American Society of Echocardiography and the European Association of Cardiovascular Imaging” [10]; secondly, given that 2013 data from the UK demonstrates that only 32.9% of men and 42.8% of women had a BMI less than 25 kg/m^2^, arguably our reference ranges represent the “new” normal range and are thus more applicable to the general population [35].

CMR examinations were not performed repeatedly on the same individuals over time, therefore the associations described between age and CMR parameters are not longitudinal, but rather cross-sectional.

## Conclusions

This study provides normal reference ranges for all four cardiac chambers derived from the largest healthy cohort of Caucasian adults and will provide utility in the analysis of CMR examinations in both clinical and research settings.

### Additional file

Additional file 1: Supplementary materials. (PDF 475 kb)

## Appendix 1

**Table 11:** Exclusion criteria

	Number (%)
Age	
> 74 years	119 (2%)
Medical conditions	
Hypertension	1382 (28%)
High cholesterol	787 (16%)
Asthma	628 (13%)
Hypothyroidism/myxoedema	322 (6%)
Diabetes	204 (4%)
Essential hypertension	130 (3%)
Angina	127 (3%)
Heart attack/myocardial infarction	104 (2%)
Deep venous thrombosis (DVT)	87 (2%)
Type 2 diabetes	83 (2%)
Atrial fibrillation	65 (1%)
Rheumatoid arthritis	58 (1%)
Stroke	58 (1%)
Emphysema/chronic bronchitis	56 (1%)
Hyperthyroidism/thyrotoxicosis	44 (1%)
Heart valve problem/heart murmur	42 (1%)
Transient ischaemic attack (TIA)	39 (1%)
Chronic obstructive airways disease/COPD	39 (1%)
Pulmonary embolism +/− DVT	38 (1%)
Iron deficiency anaemia	33 (1%)
Ulcerative colitis	31 (1%)
Heart arrhythmia	31 (1%)
Heart/cardiac problem	31 (1%)
Sleep apnoea	28 (1%)
Polymyalgia rheumatica	28 (1%)
Miscarriage	22 (0%)
Irregular heart beat	21 (0%)
Gestational hypertension/pre-eclampsia	20 (0%)
Doctor diagnosed bronchiectasis_Yes	18 (0%)
Anaemia	18 (0%)
Ankylosing spondylitis	18 (0%)
Rheumatic fever	16 (0%)
Sarcoidosis	15 (0%)
Peripheral vascular disease	14 (0%)
Bronchiectasis	14 (0%)
Diabetic eye disease	14 (0%)
Crohns disease	13 (0%)
Pernicious anaemia	11 (0%)
Gestational diabetes only_Yes	9 (0%)
Clotting disorder/excessive bleeding	9 (0%)
SVT / supraventricular tachycardia	9 (0%)
Other respiratory problems	8 (0%)
Sjogren’s syndrome/sicca syndrome	8 (0%)
Systemic lupus erythematosis/SLE	8 (0%)
Renal/kidney failure	8 (0%)
Low platelets/platelet disorder	7 (0%)
Type 1 diabetes	7 (0%)
Grave’s disease	6 (0%)
Heart failure/pulmonary edema	6 (0%)
Gestational diabetes	5 (0%)
Hereditary/genetic haematological disorder	5 (0%)
Cardiomyopathy	5 (0%)
Hyperparathyroidism	5 (0%)
Nephritis	5 (0%)
Haemochromatosis	5 (0%)
Connective tissue disorder	4 (0%)
Renal failure not requiring dialysis	4 (0%)
Polycythaemia vera	4 (0%)
Neutropenia/lymphopenia	4 (0%)
Anorexia/bulimia/other eating disorder	4 (0%)
Surgery/amputation of toe or leg_Do not know	4 (0%)
Lymphoedema	4 (0%)
Aortic stenosis	4 (0%)
Retinal artery/vein occlusion	4 (0%)
Inflammatory bowel disease	3 (0%)
Adrenocortical insufficiency/Addison’s disease	3 (0%)
Hyperprolactinaemia	3 (0%)
Surgery/amputation of toe or leg_Yes, toes	3 (0%)
Atrial flutter	3 (0%)
Mitral regurgitation/incompetence	3 (0%)
Pericarditis	3 (0%)
Hypertrophic cardiomyopathy (HCM / HOCM)	3 (0%)
Emphysema	3 (0%)
Kidney nephropathy	3 (0%)
Myocarditis	2 (0%)
Liver failure/cirrhosis	2 (0%)
Diabetic neuropathy/ulcers	2 (0%)
Leg claudication/intermittent claudication	2 (0%)
Mitral valve disease	2 (0%)
Mitral valve prolapse	2 (0%)
Monoclonal gammopathy/not myeloma	2 (0%)
Glomerulnephritis	1 (0%)
Haemophilia	1 (0%)
Vasculitis	1 (0%)
Wegners granulmatosis	1 (0%)
Sickle cell disease	1 (0%)
Microscopic polyarteritis	1 (0%)
Myositis/myopathy	1 (0%)
Pericardial problem	1 (0%)
Pleural plaques (not known asbestosis)	1 (0%)
Hyperaldosteronism/Conn’s syndrome	1 (0%)
Polymyositis	1 (0%)
Hypopituitarism	1 (0%)
Interstitial lung disease	1 (0%)
Alcoholic liver disease/alcoholic cirrhosis	1 (0%)
Antiphospholipid syndrome	1 (0%)
Aortic aneurysm	1 (0%)
Aortic regurgitation/incompetence	1 (0%)
Aplastic anaemia	1 (0%)
Diabetes insipidus	1 (0%)
Fibrosing alveolitis/unspecified alveolitis	1 (0%)
Giant cell/temporal arteritis	1 (0%)
Iga nephropathy	1 (0%)
Myeloproliferative disorder	1 (0%)
Pericardial effusion	1 (0%)
Pleural effusion	1 (0%)
Respiratory failure	1 (0%)
Sick sinus syndrome	1 (0%)
Wolff parkinson white/WPW syndrome	1 (0%)
Surgery/amputation of toe or leg_Yes, leg above the knee	1 (0%)
Surgery/amputation of toe or leg_Yes, leg below the knee	1 (0%)
Medications	
Cholesterol lowering medication	784 (16%)
Blood pressure medication	705 (14%)
Hormone replacement therapy	331 (7%)
Insulin	15 (0%)
Symptoms	
Chest pain due to walking ceases when standing still_Yes	264 (5%)
Chest pain or discomfort when walking uphill or hurrying_Yes	229 (5%)
Chest pain or discomfort when walking uphill or hurrying_Unable to walk up hills or to hurry	20 (0%)
Chest pain due to walking ceases when standing still_Do not know	17 (0%)
Chest pain or discomfort when walking uphill or hurrying_Prefer not to answer	2 (0%)
Shortness of breath walking on level ground_Yes	386 (8%)
Shortness of breath walking on level ground_Do not know	76 (2%)
Shortness of breath walking on level ground_Prefer not to answer	5 (0%)
Smoking history	
Ex-smoker	1896 (38%)
Current smoker	355 (7%)
High body mass index	
BMI ≥ 30	1158 (23%)
Ethnicity	
Other ethnic group	30 (1%)
Indian	29 (1%)
Pakistani	19 (0%)
Caribbean	19 (0%)
Chinese	17 (0%)
Prefer not to answer	17 (0%)
African	16 (0%)
Any other mixed background	15 (0%)
Any other Asian background	12 (0%)
White and Black Caribbean	8 (0%)
White and Asian	7 (0%)
White and Black African	5 (0%)
Bangladeshi	2 (0%)
Do not know	2 (0%)
Any other Black background	1 (0%)
Asian or Asian British	1 (0%)

N.B. Criteria listed are not mutually exclusive

## Appendix 2

**Table 12:** Ventricular parameters stratified by gender

	All	Males	Females
Number	800	368	432
LVEDV (ml)	143 ± 34	166 ± 32	124 ± 21
LVESV (ml)	58 ± 17	69 ± 16	49 ± 11
LVSV (ml)	85 ± 20	96 ± 20	75 ± 14
LV mass (g)	85 ± 24	103 ± 21	70 ± 13
indexed LVEDV (ml/m^2^)	79 ± 14	85 ± 15	74 ± 12
indexed LVESV (ml/m^2^)	32 ± 8	36 ± 8	29 ± 6
indexed LVSV (ml/m^2^)	47 ± 9	49 ± 10	45 ± 8
indexed LV mass (g/m^2^)	47 ± 10	53 ± 9	42 ± 7
LVEF (%)	60 ± 6	58 ± 5	61 ± 5
LV mass to volume ratio (g/ml)	0.60 ± 0.11	0.63 ± 0.11	0.57 ± 0.11
RVEDV (ml)	154 ± 40	182 ± 36	130 ± 24
RVESV (ml)	69 ± 24	85 ± 22	55 ± 15
RVSV (ml)	85 ± 20	97 ± 20	75 ± 14
indexed RVEDV (ml/m^2^)	85 ± 17	93 ± 17	77 ± 13
indexed RVESV (ml/m^2^)	38 ± 11	43 ± 11	33 ± 9
indexed RVSV (ml/m^2^)	47 ± 9	50 ± 9	45 ± 8
RVEF (%)	56 ± 6	54 ± 6	58 ± 6

The data are presented in mean ± SD. The independent sample t-test’s *p*-value was <0.0001 for all parameters

LV, left ventricle; RV, right ventricle; EDV, end-diastolic volume; ESV, end-systolic volume; SV, stroke volume; EF, ejection fraction; indexed, absolute values divided by body surface area

**Table 13:** Atrial parameters stratified by gender

	All	Males	Females
Number	795	363	432
Maximal LA volume (2Ch) (ml)*	61 ± 20	66 ± 20	57 ± 18
Maximal LA volume (4Ch) (ml)*	74 ± 22	78 ± 23	70 ± 21
Maximal LA volume (Biplane) (ml)*	66 ± 19	71 ± 19	62 ± 17
LA SV (Biplane) (ml)*	40 ± 11	42 ± 11	37 ± 10
indexed Maximal LA volume (2Ch) (ml)	34 ± 10	34 ± 10	34 ± 10
indexed Maximal LA volume (4Ch) (ml)	41 ± 12	40 ± 12	42 ± 12
indexed Maximal LA volume (Biplane) (ml)	37 ± 10	36 ± 9	37 ± 10
indexed LA SV (Biplane) (ml)	22 ± 6	22 ± 6	22 ± 6
LA EF (Biplane) (%)	60 ± 7	60 ± 7	61 ± 7
Maximal RA volume (4Ch) (ml)*	80 ± 25	93 ± 27	69 ± 17
RA SV (4Ch) (ml)*	35 ± 13	38 ± 14	32 ± 10
indexed Maximal RA volume (4Ch) (ml)*	44 ± 12	48 ± 14	41 ± 10
indexed RA SV (4Ch) (ml)	19 ± 7	20 ± 7	19 ± 6
RA EF (4Ch) (%)*	44 ± 10	41 ± 9	46 ± 9

The data are presented in mean ± SD. **p*-value < 0.0001

LA, left atrium; RA, right atrium; SV, stroke volume; EF, ejection fraction; 2Ch, two-chamber; 4Ch, four-chamber; Biplane, derived from four-chamber and two-chamber views; indexed, absolute values divided by body surface area

**Table 14:** Correlation table for ventricular parameters with age

	Males		Females	
	*r* ^a^	Level of significance	*r* ^a^	Level of
Significance				
LVEDV	−0.19	****	−0.19	****
LVESV	−0.14	**	−0.16	**
LVSV	−0.18	****	−0.16	**
LV mass	−0.13	*	−0.04	
indexed LVEDV	−0.13	*	−0.15	**
indexed LVESV	−0.09		−0.13	*
indexed LVSV	−0.12	*	−0.12	*
indexed LV mass	−0.07		0.01	
LVEF	−0.02		0.03	
LV mass to volume ratio	0.06		0.14	**
RVEDV	−0.21	****	−0.18	****
RVESV	−0.18	****	−0.18	****
RVSV	−0.19	****	−0.12	*
indexed RVEDV	−0.16	**	−0.15	**
indexed RVESV	−0.14	**	−0.16	**
indexed RVSV	−0.13	*	−0.08	
RVEF	0.06		0.11	*

**** *p* < 0.001; *** *p* < 0.001; ** *p* < 0.01; * *p* < 0.05

^a^Pearson correlation coefficient

LV, left ventricle; RV, right ventricle; EDV, end-diastolic volume; ESV, end-systolic volume; SV, stroke volume; EF, ejection fraction; indexed, absolute values divided by body surface area

**Table 15:** Correlation table for atrial parameters with age

	Males		Females	
	*r* ^a^	Level of significance	*r* ^a^	Level of significance
Maximal LA volume (2Ch)	−0.11	*	−0.11	*
Maximal LA volume (4Ch)	−0.1		−0.14	**
Maximal LA volume (Biplane)	−0.11	*	−0.14	**
LA SV (Biplane)	−0.12	*	−0.23	****
indexed Maximal LA volume (2Ch)	−0.07		−0.08	
indexed Maximal LA volume (4Ch)	−0.05		−0.11	*
indexed Maximal LA volume (Biplane)	−0.06		−0.11	*
indexed LA SV (Biplane)	−0.07		−0.2	****
LA EF (Biplane)	−0.01		−0.15	**
Maximal RA volume (4Ch)	0.01		0	
RA SV (4Ch)	0		−0.06	
indexed Maximal RA volume (4Ch)	0.06		0.04	
indexed RA SV (4Ch)	0.04		−0.03	
RA EF (4Ch)	0.01		−0.11	*

**** *p* < 0.001; *** *p* < 0.001; ** *p* < 0 .01; * *p* < 0.05

^a^Pearson correlation coefficient

LA, left atrium; RA, right atrium; SV, stroke volume; EF, ejection fraction; 2Ch, two-chamber; 4Ch, four-chamber; Biplane, derived from four-chamber and two-chamber views; indexed, absolute values divided by body surface area

## Appendix 4

### UK Biobank data

UK Biobank data in a codified tabular format, received through our access application, was used to select the healthy cohort. Data was translated, using the data dictionary provided as part of the application and the coding tables available through the UK Biobank website, into a self-contained table which we used to perform the analysis. The data derived from the analysis of CMR studies were tested for gross errors such as non-physiological values (e.g., end-systolic volume larger than end-diastolic volume) and were removed from the final dataset.

## Abbreviations

[b]SSFP: [Balanced] steady state free precession
BMI: Body mass index
BSA: Body surface area
CMR: Cardiovascular magnetic resonance
HLA: Horizontal long axis
ICC: Intra-class correlation coefficient
LA: Left atrium
LV: Left ventricle
LVOT: Left ventricular outflow tract
MESA: Multi-Ethnic Study of Atherosclerosis
RA: Right atrium
RV: Right ventricle
SD: Standard deviation
TE: Echo time
TR: Repetition time
VLA: Vertical long axis

## References

[1] Maceira AM, Prasad SK, Khan M, Pennell DJ (2006). Normalized left ventricular systolic and diastolic function by steady state free precession cardiovascular magnetic resonance. J Cardiovasc Magn Reson.

[2] Hudsmith L, Petersen S, Francis J, Robson M, Neubauer S (2005). Normal human left and right ventricular and left atrial dimensions using steady state free precession magnetic resonance imaging. J Cardiovasc Magn Reson.

[3] Alfakih K, Plein S, Thiele H, Jones T, Ridgway JP, Sivananthan MU. Normal human left and right ventricular dimensions for MRI as assessed by turbo gradient echo and steady-state free precession imaging sequences. J Magn Reson Imaging. 2003;17:323–9. 10.1002/jmri.10262 12594722

[4] Kawel-Boehm N, Maceira A, Valsangiacomo-Buechel ER, Vogel-Claussen J, Turkbey EB, Williams R (2015). Normal values for cardiovascular magnetic resonance in adults and children. J Cardiovasc Magn Reson BioMed Central.

[5] Yeon SB, Salton CJ, Gona P, Chuang ML, Blease SJ, Han Y (2015). Impact of age, sex, and indexation method on MR left ventricular reference values in the Framingham Heart Study offspring cohort. J Magn Reson Imaging NIH Public Access.

[6] Foppa M, Arora G, Gona P, Ashrafi A, Salton CJ, Yeon SB (2016). Right ventricular volumes and systolic function by cardiac magnetic resonance and the impact of sex, age, and obesity in a longitudinally followed cohort free of pulmonary and cardiovascular disease. Circ Cardiovasc Imaging Lippincott Williams & Wilkins.

[7] Sudlow C, Gallacher J, Allen N, Beral V, Burton P, Danesh J (2015). UK Biobank: an open access resource for identifying the causes of a wide range of complex diseases of middle and old age. PLoS Med Public Library of Science.

[8] Petersen SE, Matthews PM, Bamberg F, Bluemke DA, Francis JM, Friedrich MG (2013). Imaging in population science: cardiovascular magnetic resonance in 100,000 participants of UK Biobank - rationale, challenges and approaches. J Cardiovasc Magn Reson.

[9] Petersen SE, Matthews PM, Francis JM, Robson MD, Zemrak F, Boubertakh R (2016). UK Biobank’s cardiovascular magnetic resonance protocol. J Cardiovasc Magn Reson BioMed Central Ltd.

[10] Lang RM, Badano LP, Mor-Avi V, Afilalo J, Armstrong A, Ernande L (2015). Recommendations for cardiac chamber quantification by echocardiography in adults: an update from the American Society of Echocardiography and the European Association of Cardiovascular Imaging. J Am Soc Echocardiogr.

[11] Carapella V, Jimenez-Ruiz E, Lukaschuk E, Aung N, Fung K, Paiva J, et al. Towards the semantic enrichment of free-text annotation of image quality assessment for UK Biobank cardiac Cine MRI scans. Lect Notes Comput Sci. 2016;238–48. 1.

[12] Solberg HE (1983). The theory of reference values Part 5. Statistical treatment of collected reference values. Determination of reference limits. J. Clin. Chem. Clin. Biochem. Zeitschrift für Klin. Chemie und Klin. Biochem..

[13] Du Bois D, Du Bois EF (1916). A formula to estimate the approximate surface area if height and weight be known. Arch Intern Med.

[14] Bland JM, Altman DG (1986). Statistical methods for assessing agreement between two methods of clinical measurement. Lancet (London, England).

[15] Hallgren KA (2012). Computing inter-rater reliability for observational data: an overview and tutorial. Tutor Quant Methods Psychol NIH Public Access.

[16] R Core Team. R: A Language and Environment for Statistical Computing. Vienna; 2016.

[17] Dweck MR, Joshi S, Murigu T, Gulati A, Alpendurada F, Jabbour A (2012). Left ventricular remodeling and hypertrophy in patients with aortic stenosis: insights from cardiovascular magnetic resonance. J Cardiovasc Magn Reson.

[18] Sievers B, Kirchberg S, Franken U, Bakan A, Addo M, John-Puthenveettil B (2005). Determination of normal gender-specific left atrial dimensions by cardiovascular magnetic resonance imaging. J Cardiovasc Magn Reson.

[19] Maceira AM, Cosín-Sales J, Roughton M, Prasad SK, Pennell DJ (2010). Reference left atrial dimensions and volumes by steady state free precession cardiovascular magnetic resonance. J Cardiovasc Magn Reson.

[20] Maceira AM, Cosín-Sales J, Roughton M, Prasad SK, Pennell DJ, Sanfilippo A (2013). Reference right atrial dimensions and volume estimation by steady state free precession cardiovascular magnetic resonance. J Cardiovasc Magn Reson BioMed Central.

[21] Gandy SJ, Lambert M, Belch J, Cavin I, Crowe E, Littleford R, et al. 3T MRI investigation of cardiac left ventricular structure and function in a UK population: The tayside screening for the prevention of cardiac events (TASCFORCE) study. J Magn Reson Imaging. 2016;44:1186–96. 10.1002/jmri.25267 PMC5082537 27143317

[22] Le Ven F, Bibeau K, De Larochellière É, Tizón-Marcos H, Deneault-Bissonnette S, Pibarot P, et al. Cardiac morphology and function reference values derived froma large subset of healthy young Caucasian adults by magnetic resonance imaging. Eur Heart J Cardiovasc Imaging. 2016;17:981–90. 10.1093/ehjci/jev217 PMC5066336 26354980

[23] Helge Erik Solberg DS (1991). IFCC recommendation: the theory of reference values. Part 4. Control ofanalytical variation in the production, transfer and application of reference values. J Automat Chem Hindawi Publishing Corporation.

[24] Lieb W, Xanthakis V, Sullivan LM, Aragam J, Pencina MJ, Larson MG (2009). Longitudinal tracking of left ventricular mass over the adult life course: clinical correlates of short- and long-term change in the framingham offspring study. Circulation American Heart Association Journals.

[25] Fuchs A, Mejdahl MR, Kühl JT, Stisen ZR, Nilsson EJP, Køber LV (2016). Normal values of left ventricular mass and cardiac chamber volumes assessed by 320-detector computed tomography angiography in the Copenhagen General Population Study. Eur Hear. J Cardiovasc Imaging. Oxford University Press.

[26] Chung AK, Das SR, Leonard D, Peshock RM, Kazi F, Abdullah SM (2006). Women have higher left ventricular ejection fractions than men independent of differences in left ventricular volume: the Dallas Heart Study. Circulation American Heart Association Journals.

[27] Natori S, Lai S, Finn JP, Gomes AS, Hundley WG, Jerosch-Herold M, et al. Cardiovascular Function in Multi-Ethnic Study of Atherosclerosis: Normal Values by Age, Sex, and Ethnicity. Am J Roentgenol. American Roentgen Ray Society; 2012. 10.2214/AJR.04.1868 16714609

[28] Strait JB, Lakatta EG (2012). Aging-associated cardiovascular changes and their relationship to heart failure. Heart Fail Clin NIH Public Access.

[29] Schulman SP, Lakatta EG, Fleg JL, Lakatta L, Becker LC, Gerstenblith G (1992). Age-related decline in left ventricular filling at rest and exercise. Am J Physiol.

[30] Kitzman DW, Scholz DG, Hagen PT, Ilstrup DM, Edwards WD (1988). Age-related changes in normal human hearts during the first 10 decades of life. Part II (maturity): a quantitative anatomic study of 765 specimens from subjects 20 to 99 years old. Mayo Clin Proc.

[31] Margossian R, Schwartz ML, Prakash A, Wruck L, Hurwitz LM, Marcus E (2010). Comparison of echocardiographic and cardiac magnetic resonance imaging measurements of functional single ventricular volumes, mass, and ejection fraction (From the Pediatric Heart Network Multicenter Fontan Cross-Sectional Study). Am J Cardiol.

[32] Teo KSL, Carbone A, Piantadosi C, Chew DP, Hammett CJK, Brown MA (2008). Cardiac MRI assessment of left and right ventricular parameters in healthy Australian normal volunteers. Hear Lung Circ.

[33] Rider OJ, Francis JM, Ali MK, Byrne J, Clarke K, Neubauer S (2009). Determinants of left ventricular mass in obesity; a cardiovascular magnetic resonance study. J Cardiovasc Magn Reson BioMed Central.

[34] Rider OJ, Petersen SE, Francis JM, Ali MK, Hudsmith LE, Robinson MR (2011). Ventricular hypertrophy and cavity dilatation in relation to body mass index in women with uncomplicated obesity. Heart. BMJ Publishing Group Ltd and British Cardiovascular Society.

[35] Health and Social Care Information Centre. Statistics on Obesity, Physical Activity and Diet. 2016.

