# Supplementary material for: Reference ranges for cardiac structure and function using cardiovascular magnetic resonance (CMR) in Caucasians from the UK Biobank population cohort
Source: J Cardiovasc Magn Reson. 2017 Feb 3;19:18. doi: 10.1186/s12968-017-0327-9 (PMC5304550; doi:10.1186/s12968-017-0327-9)
Supplement: Supplementary file 1 — Supplementary materials. (PDF 475 kb) [file 12968_2017_327_MOESM1_ESM.pdf]

CMR REFERENCE RANGES FOR AGES 45-74: DATA FROM THE UK BIOBANK

	MALE					FEMALE				
	Abnormal low	Borderline zone*	Normal zone	Borderline zone*	Abnormal high	Abnormal low	Borderline zone*	Normal zone	Borderline zone*	Abnormal high
**Left ventricle**										
LVEDV (ml)	<93		109 - 218		>232	<80		88 - 161		>175
LVESV (ml)	<34		39 - 97		>103	<25		31 - 68		>73
LVSV (ml)	<49		59 - 132		>140	<47		49 - 100		>110
LV mass (g)	<56		64 - 141		>148	<44		46 - 93		>96
indexed LVEDV (ml/m^2)	<52		60 - 110		>117	<50		54 - 94		>101
indexed LVESV (ml/m^2)	<19		21 - 49		>52	<16		19 - 40		>43
indexed LVSV (ml/m^2)	<28		32 - 67		>70	<29		30 - 59		>63
indexed LV mass (g/m^2)	<33		35 - 70		>72	<28		29 - 55		>55
LVEF (%)	<47		48 - 69		>70	<50		51 - 70		>72
LV mass to volume ratio (g/ml)	<0.40		0.42 - 0.84		>0.87	<0.35		0.39 - 0.71		>0.81
**Right ventricle**										
RVEDV (ml)	<99		124 - 248		>260	<83		85 - 168		>192
RVESV (ml)	<34		47 - 123		>135	<26		27 - 77		>95
RVSV (ml)	<54		62 - 131		>140	<47		48 - 99		>107
indexed RVEDV (ml/m^2)	<55		68 - 125		>128	<51		53 - 99		>110
indexed RVESV (ml/m^2)	<19		25 - 63		>67	<16		17 - 46		>55
indexed RVSV (ml/m^2)	<30		34 - 67		>69	<29		30 - 59		>61
RVEF (%)	<40		45 - 65		>68	<45		47 - 68		>70

	MALE					FEMALE				
	Abnormal low	Borderline zone*	Normal zone	Borderline zone*	Abnormal high	Abnormal low	Borderline zone*	Normal zone	Borderline zone*	Abnormal high
**Left atrium**										
Max. LA vol (2Ch) (ml)	<22		30 - 104		>112	<22		30 - 104		>112
Max. LA vol (4Ch) (ml)	<23		36 - 124		>125	<23		36 - 124		>125
Max. LA vol (Biplane) (ml)	<26		37 - 108		>112	<26		37 - 108		>112
LA SV (Biplane) (ml)	<16		23 - 62		>66	<16		23 - 62		>66
indexed Max. LA vol (2Ch) (ml/m^2)	<12		16 - 53		>56	<12		16 - 53		>56
indexed Max. LA vol (4Ch) (ml/m^2)	<14		19 - 62		>63	<14		19 - 62		>63
indexed Max LA vol (Biplane) (ml/m^2)	<15		19 - 55		>56	<15		19 - 55		>56
indexed LA SV (Biplane) (ml/m^2)	<9		12 - 32		>33	<9		12 - 32		>33
LA EF (Biplane) (%)	<44		47 - 73		>75	<44		47 - 73		>75
**Right atrium**										
Max. RA vol (4Ch) (ml)	<36		43 - 143		>150	<36		43 - 143		>150
RA SV (4Ch) (ml)	<9		10 - 66		>66	<9		10 - 66		>66
indexed Max. RA vol (4Ch) (ml/m^2)	<19		22 - 74		>79	<19		22 - 74		>79
indexed RA SV (4Ch) (ml/m^2)	<5		5 - 33		>35	<5		5 - 33		>35
RA EF (4Ch) (%)	<21		23 - 58		>60	<21		23 - 58		>60

Data described in this table can be found in the paper entitled "Reference ranges for cardiac structure and function using cardiovascular magnetic resonance (CMR) in Caucasians from the UK Biobank population cohort" by Petersen et al. in the Journal of Cardiovascular Magnetic Resonance, 2017. *DOI 10.1186/s12968-017-0327-9*
Borderline zone data can be found in the main manuscript.
